# Development of a core outcome set (COS) for studies relating to awareness and clinical management of reduced fetal movement: study protocol

**DOI:** 10.1186/s13063-021-05839-9

**Published:** 2021-12-09

**Authors:** Dexter J. L. Hayes, Declan Devane, Jo C. Dumville, Valerie Smith, Tanya Walsh, Alexander E. P. Heazell

**Affiliations:** 1 Tommy’s Stillbirth Research Centre, School of Medical Sciences, Division of Developmental Biology and Medicine, Faculty of Biology, Medicine, and Health, University of Manchester, Manchester, UK; 2 HRB-Trials Methodology Research Network, School of Nursing and Midwifery, National University of Ireland, Galway, Ireland; 3 Division of Nursing, Midwifery and Social Work, School of Health Sciences, Faculty of Biology, Medicine, and Health, University of Manchester, Manchester Academic Science Centre, Manchester, UK; 4 School of Nursing & Midwifery, Trinity College Dublin, The University of Dublin, Dublin, Ireland; 5 School of Dentistry, University of Manchester, Manchester, UK

**Keywords:** Core outcome set, Pregnancy, Reduced fetal movement

## Abstract

**Background:**

Concerns regarding reduced fetal movements (RFM) are reported in 5–15% of pregnancies, and RFM are associated with adverse pregnancy outcomes including fetal growth restriction and stillbirth. Studies have aimed to improve pregnancy outcomes by evaluating interventions to raise awareness of RFM in pregnancy, such as kick counting, evaluating interventions for the clinical management of RFM, or both. However, there is not currently a core outcome set (COS) for studies of RFM. This study aims to create a COS for use in research studies that aim to raise awareness of RFM and/or evaluate interventions for the clinical management of RFM.

**Methods:**

A systematic review will be conducted, to identify outcomes used in randomised and non-randomised studies with control groups that aimed to raise awareness of RFM (for example by using mindfulness techniques, fetal movement counting, or other tools such as leaflets or mobile phone applications) and/or that evaluated the clinical management of RFM.

An international Delphi consensus will then be used whereby stakeholders will rate the importance of the outcomes identified in the systematic review in (i) awareness and (ii) clinical management studies. The preliminary lists of outcomes will be discussed at a consensus meeting where one final COS for awareness and management, or two discrete COS (one for awareness and one for management), will be agreed upon.

**Discussion:**

A well-developed COS will provide researchers with the minimum set of outcomes that should be measured and reported in studies that aim to quantify the effects of interventions.

**Supplementary Information:**

The online version contains supplementary material available at 10.1186/s13063-021-05839-9.

## Background

### Reduced fetal movement and adverse pregnancy outcome

Reduced fetal movements (RFM) are usually defined as a subjective decrease or change in a baby’s typical pattern of movements in utero [1]; current guidance in the UK and Australia is for anyone pregnant to contact a midwife or maternity unit if their baby is moving less than usual or not at all [2, 3]. Most pregnant people become aware of fetal movements by 18–20 weeks’ gestation, the pattern of their baby’s movements, and the time of day that the baby moves the most by 28 weeks’ gestation [4]. Awareness of fetal activity is recognised as one component of maternal-fetal attachment [5].

Maternal concern regarding RFM leads to a presentation at the hospital in 5–15% of pregnancies [6]. Around 70% of these pregnancies have a normal outcome [7–9], but observational studies have recurrently demonstrated that RFM are associated with adverse pregnancy outcomes, including fetal growth restriction (FGR) and stillbirth, supporting the potential for a common aetiology [10, 11]. Case-control studies have consistently demonstrated an association between reduced frequency and strength of fetal movements and stillbirth after 28 weeks’ gestation [12–14]; this effect has also been seen in low-income settings [15]. It is thought that RFM may be an attempt by the fetus to conserve energy and oxygen consumption in cases of insufficient nutrient transfer and hypoxia, which in turn may be caused by placental insufficiency or other fetal stressors [16–18].

### Studies of awareness of or interventions for reduced fetal movement

Studies have aimed to improve pregnancy outcomes by evaluating interventions that raise maternal and/or clinical awareness of RFM, such as mindfulness or kick counting [19, 20], and/or by evaluating clinical management interventions, for example, interventions comprised of further monitoring and/or clinical testing such as cardiotocography (CTG) or ultrasound (US) to identify whether RFM is an indicator of an underlying condition that may warrant further clinical intervention or even expedited birth [21, 22].

Despite the association between RFM, stillbirth and FGR, a core outcome set (COS) for studies evaluating interventions that aim to raise awareness of RFM and/or studies evaluating the clinical management of RFM does not currently exist. This means that studies often measure and report different outcomes and employ different definitions for these outcomes, hindering meta-analysis of studies. A COS describes a standardised set of outcomes that should be measured and reported in all studies in a specific area as a minimum [23]; COS are currently in use across several healthcare fields, including maternity care [24–26]. It is anticipated that developing a COS will ensure that the most important and relevant outcomes, as agreed by stakeholder consensus, are measured, thus optimising the synthesis of individual studies. This will further facilitate data synthesis and interpretation of the evidence based on prioritised outcomes.

## Methods

### Aim

This protocol describes the development of a COS for measurement and reporting in studies that aim to raise awareness of RFM and/or evaluate the clinical management of RFM. Development and adoption of this COS will ensure consistent and relevant outcome measurement and reporting in studies for raising awareness and/or evaluating the clinical management of RFM, which may lead to more robust results, improved wellbeing in pregnancy, and may also be applicable in clinical practice.

This COS will apply to controlled randomised and non-randomised study designs, as described in Table 1.

**Table 1:** Study inclusion criteria for the systematic review

Population	Singleton pregnancies presenting at least once in a maternity care setting after 28 weeks’ gestation
Intervention	Any intervention aimed at raising awareness of RFM and/or evaluating the clinical management of RFM
Comparator	Any other intervention described above or no intervention
Outcome	Any maternal or fetal outcomes
Study design	Controlled randomised and non-randomised studies with clearly reported mechanism of group formation, clearly defined inclusion criteria, and described methods of ascertainment of eligible patients and their recruitment

### Objectives

1. To systematically review the outcomes included in intervention studies for raising awareness of RFM and/or evaluating its clinical management
2. To develop a consensus on a preliminary COS using these outcomes via the Delphi survey
3. To develop a final COS for use in all future intervention studies aimed at raising awareness of RFM and/or evaluating the clinical management of RFM, via an international consensus meeting with key stakeholders
4. To disseminate and promote the use of the COS

### Design

The COS development project was registered with the Core Outcome Measures in Effectiveness Trials (COMET) initiative (http://comet-initiative.org/Studies/Details/928) on the 24th of September 2020.

This protocol was developed in accordance with the COMET handbook [23]. A study steering committee will be established comprised of at least one representative from the following relevant stakeholder groups: researchers, clinicians, and patients (described in more detail later). We will include at least one representative from low- or middle-income countries on the steering committee.

### Stage 1: Systematic review

A systematic review of the literature will be conducted to identify outcomes measured in studies of interventions where the intervention is designed to raise awareness of RFM and/or evaluate the clinical management of RFM.

The target population is women with non-anomalous singleton pregnancies after 28 weeks’ gestation; this threshold has been chosen over other definitions of stillbirth in order to facilitate international comparisons [27]. We will not include studies of multiple pregnancies because observational data about the association between RFM and adverse outcome in this group is less clear.

We will include controlled randomised and non-randomised studies with clearly reported mechanisms of group formation, clearly defined inclusion criteria, and clearly described methods of ascertainment of eligible patients and their recruitment. Studies will be included regardless of their publication status and language of publication.

Pregnancy, labour, and birth outcomes will be extracted with their corresponding definitions where possible. Outcomes will be grouped as maternal or neonatal, and different definitions of the same outcome will be grouped into single outcome measures. This will be facilitated by discussions between members of the study team. This final list of outcomes will be used in stage two.

We will search the following databases: MEDLINE, MEDLINE (In-Process and Other Non-Indexed Citations), Embase, EBSCO CINAHL Plus, the Cochrane Central Register of Controlled Trials (CENTRAL), the Cochrane Pregnancy and Childbirth’s Trials Register, and the Cochrane Database of Systematic Reviews. Other trial registries such as clinicaltrials.gov, WHO ICTRP, and the EU clinical trials register will also be searched, as well as databases such as OpenGrey (www.opengrey.eu), Joanna Briggs Institute (www.joannabriggs.edu.au), and the National Institute for Health and Clinical Excellence website (NICE; www.nice.org.uk) to find unpublished studies. Reference lists of included papers will be reviewed for additional studies. The search strategy for this COS is provided as an additional file.

Studies identified by the literature searches will be independently screened for inclusion by two study authors using our study inclusion criteria (Table 1). Disagreements will be resolved by consulting a third author. The following data will be extracted from included studies: study aim, location, population, setting, description of the intervention and comparator, and outcomes reported in the study.

### Stage 2: Online international Delphi survey

A sequential three-round electronic international Delphi study will be conducted using REDCap v10.1.2 including key stakeholders to produce a preliminary COS. Each round will remain open for at least 14 days, and a reminder email will be sent out three working days before closure. Data from each round will be analysed and presented to participants in the next round (described in more detail below). Attrition rates for each round will also be assessed. All participants’ contact information will remain confidential.

The Delphi survey and following consensus meeting will allow the possibility of producing either one COS (for all studies relating to raising awareness and/or evaluating the clinical management of RFM) or two COS (one for studies raising awareness of RFM and another for studies evaluating the clinical management of RFM). This will be dependent on whether there is significant overlap or similarity in the final outcomes in the two lists. This follows the precedent set by other COS in maternity care that have started by running two surveys simultaneously and then voted on whether one COS should be produced [26, 28].

#### Participants

We will invite people from all stakeholder groups, aiming for at least 15 people from each of the following three groups to ensure adequate representation. Eligible participants will be (1) researchers, research funders, and policymakers who are actively involved in work related to RFM; (2) clinicians (midwives, obstetricians, neonatologists, GPs/family physicians) with experience of caring for women with RFM; and (3) anyone who is or who has been pregnant and their partners if applicable. We will recruit participants through professional organisations, electronic discussion lists, and patient organisations or charities. As with the formation of the steering group, we will ensure that participants from low-income countries are represented. Authors of all included studies will be invited to participate; we will also use snowball sampling, whereby we will request participants to forward the survey to others who they consider eligible to participate.

Participants of the Delphi survey will receive all information regarding the study as part of the invitation email. Consent to take part in the survey will be ensured by requiring participants to click an ‘I agree to take part’ box before gaining access to the survey. All personal data of participants will only be accessible to members of the research team and any response to the survey will be confidential. Participants will have the right to withdraw at any point.

#### Round 1

Round 1 will collect demographic data including nationality, age, stakeholder group, and role. Participants will be presented with a list of all outcomes identified from the systematic review and will be asked to rate the importance of each using a 9-point Likert scale. On this scale, a score of 1–3 indicates limited importance, 4–6 signifies importance, and 7–9 is used for critically important outcomes. Participants will also be prompted to add additional outcomes that they feel are important but are not included in the preliminary list. Suggested outcomes will be included in round 2 if they are mentioned by at least two participants. All outcomes from round 1 will be forwarded to round 2.

Feedback will be provided to all who participated in round 1 in the form of descriptive statistics and graphical representations for ease of interpretation. For each outcome, participants will receive their scores from the first round and a graphical representation of the percentages of each group who voted for each score for each outcome. All feedback provided to participants will be anonymised.

Standardised consensus criteria will be applied to the results from this round, which will be used through all three rounds to reach the preliminary list of outcomes to be included (Table 2). Outcomes that are not scored by participants will not be included in analyses or consensus definitions.

**Table 2:** Consensus criteria for outcomes

Definition	Criteria
Consensus in	Scored as 7–9 by 70% or more of all participants, including at least one from each stakeholder group, and as 1–3 by less than 15% of participants
Consensus out	Scored as 1–3 by over 70% of participants and as 7–9 by less than 15%
No consensus	Any other combination of scores

#### Round 2

Participants will be presented with their scores from the previous round as well as feedback given in the same way as described in round 1. All participants who completed the first round will be asked to re-score all outcomes using the same 9-point Likert scale, including any additional suggested outcomes from round 1, in light of their and others’ ratings.

In round 2, outcomes will be presented in two lists. Participants will be asked to rate the outcomes in each list according to how important they think each outcome is for measuring and reporting in studies of (i) interventions for raising awareness of RFM and in studies of (ii) interventions for the clinical management of RFM. The ratings for the two separate lists will be reviewed and analysed separately.

Outcomes will be included in round 3 if they are rated as ‘consensus in’ or ‘no consensus’ using the consensus criteria in Table 2; those rated as ‘consensus out’ will be removed. Outcomes will be removed from lists (i) and (ii) of the survey individually based on their ratings in each list. We will also assess the rates of attrition from each round and whether participants change their scores based on the feedback they receive.

#### Round 3

Round 3 will only include participants who completed round 2. Participants will again be provided with feedback and asked to re-rate the outcomes retained from round 2 in the same way as in round 2, in two separate lists for (i) RFM awareness studies and (ii) RFM clinical management studies using the 9-point Likert scale. The consensus criteria will again be used to determine which outcomes are retained in each distinct outcome list following this round and forwarded to the consensus meeting. Those defined as ‘consensus out’ and ‘no consensus’ will be removed. Round 3 will include a question asking if participants are willing to take part in the final consensus meeting and if they consent to being contacted.

### Stage 3: Consensus meeting

A consensus meeting with an international panel representing views of all key stakeholders will be held to discuss, vote, and agree on the final RFM COS. This meeting will include a presentation of the findings of the round 3 Delphi, including the final list of outcomes by category (i.e. awareness and clinical management) and how they were voted for by each stakeholder group. This will be followed by a timed discussion and a vote on each outcome for each list. Outcomes will be included if voted for by at least 70% of participants, including at least one from each stakeholder group.

The final COS for (i) interventions for raising awareness of RFM and (ii) interventions for the clinical management of RFM will then be compared. If the outcomes included in these two COS lists are largely the same, then a majority vote will be held on whether to combine the lists to produce one COS for studies of both the awareness and clinical management of RFM. If the outcomes in the lists are different, then two separate COS will be created.

The consensus panel will be comprised of at least three representatives from each stakeholder group described earlier. The meeting will be held in English and group facilitators will be used for discussions. Due to the COVID-19 pandemic and in order to facilitate international attendance, we are planning that this meeting will be online.

### Stage 4: Dissemination

The final COS will be published in an open-access journal. After publication, it will be made available through the COMET and CoRe Outcomes in Women’s and Newborn health (CROWN) databases. In addition, we plan to disseminate the COS at national and international conferences and through relevant professional and patient organisations. We will share the COS with clinical trial registries and relevant consumer groups such as maternity service users and will ask all participants to share as they see appropriate.

## Discussion

Research into the management of RFM was identified by the Stillbirth Priority Setting Partnership (PSP) which prioritised the question ‘Which investigations identify a fetus which is at risk of stillbirth after a mother believes she has experienced reduced fetal movements?’ [29]. The PSP had extensive input from researchers, clinicians, stakeholder groups, and bereaved parents. However, there is currently no published COS for studies that evaluate interventions to raise awareness of RFM and/or for the clinical management of RFM. A well-developed COS for research studies that aim to quantify the effects of these interventions will provide researchers with a minimum set of outcomes that should be recorded, facilitating comparisons of interventions. We have taken steps to ensure that pregnant women and their views are adequately represented in this study and the final COS.

## Trial status

Protocol version 1.1, 4th November 2021. Recruitment for the first round of the Delphi survey has begun.

## Supplementary Information

**Additional file 1:** Core Outcome Set-STAndardised Protocol Items: the COS-STAP Statement

## Data Availability

Only the study authors will have access to the final dataset.

## Abbreviations

COS: Core outcome set
RFM: Reduced fetal movements
