# Supplementary material for: Development of a core outcome set (COS) for studies relating to awareness and clinical management of reduced fetal movement: study protocol
Source: Trials. 2021 Dec 9;22:894. doi: 10.1186/s13063-021-05839-9 (PMC8655489; doi:10.1186/s13063-021-05839-9)
Supplement: Supplementary file 1 — Additional file 1: Core Outcome Set-STAndardised Protocol Items: the COS-STAP Statement [file 13063_2021_5839_MOESM1_ESM.docx]

| Core Outcome Set-STAndardised Protocol Items: the COS-STAP Statement | | | |
| --- | --- | --- | --- |
| TITLE/ABSTRACT |  |  | Page/section |
| Title | 1a | Identify in the title that the paper describes a protocol for the planned development of a COS | Title |
| Abstract | 1b | Provide a structured abstract | Abstract, p2, line 16 |
| INTRODUCTION |  |  |  |
| Background and objectives | 2a | Describe the background and explain the  rationale for developing the COS, and identify the reasons why a COS is needed and the potential barriers to its implementation | Background, p3 |
|  | 2b | Describe the specific objectives with reference to developing a COS | Objectives, p4, lines 77-85 |
| Scope | 3a | Describe the health condition(s) and  population(s) that will be covered by the COS | Scope, p5, lines 95-106 |
|  | 3b | Describe the intervention(s) that will be covered by the COS | p5, lines 114-120 |
|  | 3c | Describe the context of use for which the COS is to be applied | P5, lines 98-102 |
| METHODS |  |  |  |
| Stakeholders | 4 | Describe the stakeholder groups to be involved in the COS development process, the nature of and rationale for their involvement and also how the individuals will be identified; this should cover involvement both as members of the research team and as participants in the study | p6, lines 151-160 |
| Information sources | 5a | Describe the information sources that will be used to identify the list of outcomes. Outline the methods or reference other protocols/papers | P6, lines 130-137 |
|  | 5b | Describe how outcomes may be dropped/ combined, with reasons | p6, lines 121-125 |
| Consensus process | 6 | Describe the plans for how the consensus process will be undertaken | p8, lines 167-199 |
| Consensus definition | 7a | Describe the consensus definition | P8, lines 178-182 |
|  | 7b | Describe the procedure for determining how outcomes will be added/combined/dropped from consideration during the consensus process | P8, lines 167-200 |
| ANALYSIS |  |  |  |
|  | 8 | Describe how outcomes will be scored and summarised, describe how participants will receive feedback during the consensus process | P8, lines 167-177 |
|  | 9 | Describe how missing data will be handled during the consensus process | P9, lines 183-184 |
| ETHICS and DISSEMINATION | | | |
| Ethics approval/ informed consent | 10 | Describe any plans for obtaining research ethics  committee/institutional review board approval in relation to the consensus process and describe how informed consent will be obtained (if relevant) | P11, lines 236-238 |
| Dissemination | 11 | Describe any plans to communicate the results to study participants and COS users, inclusive of methods and timing of dissemination | P10, lines 213-218 |
| ADMINISTRATIVE INFORMATION | | | |
| Funders | 12 | Describe sources of funding, role of funders | p11, lines 246-248 |
| Conflicts of interest | 13 | Describe any potential conflicts of interest within the study team and how they will be managed | p11, line 244 |
